# Supplementary material for: Effects of soluble electron shuttles on microbial iron reduction and methanogenesis
Source: Appl Environ Microbiol. 2025 Apr 25;91(5):e02222-24. doi: 10.1128/aem.02222-24 (PMC12093953; doi:10.1128/aem.02222-24)

**Supplemental Figure S1.** Iron(II) production, acetate consumption, or methane production in each experimental treatment visualized after three different phases of incubation - first two weeks of primarily iron reduction, middle two weeks transition from iron reduction to methane production, and remainder 32 days when acetate was completely consumed.

**
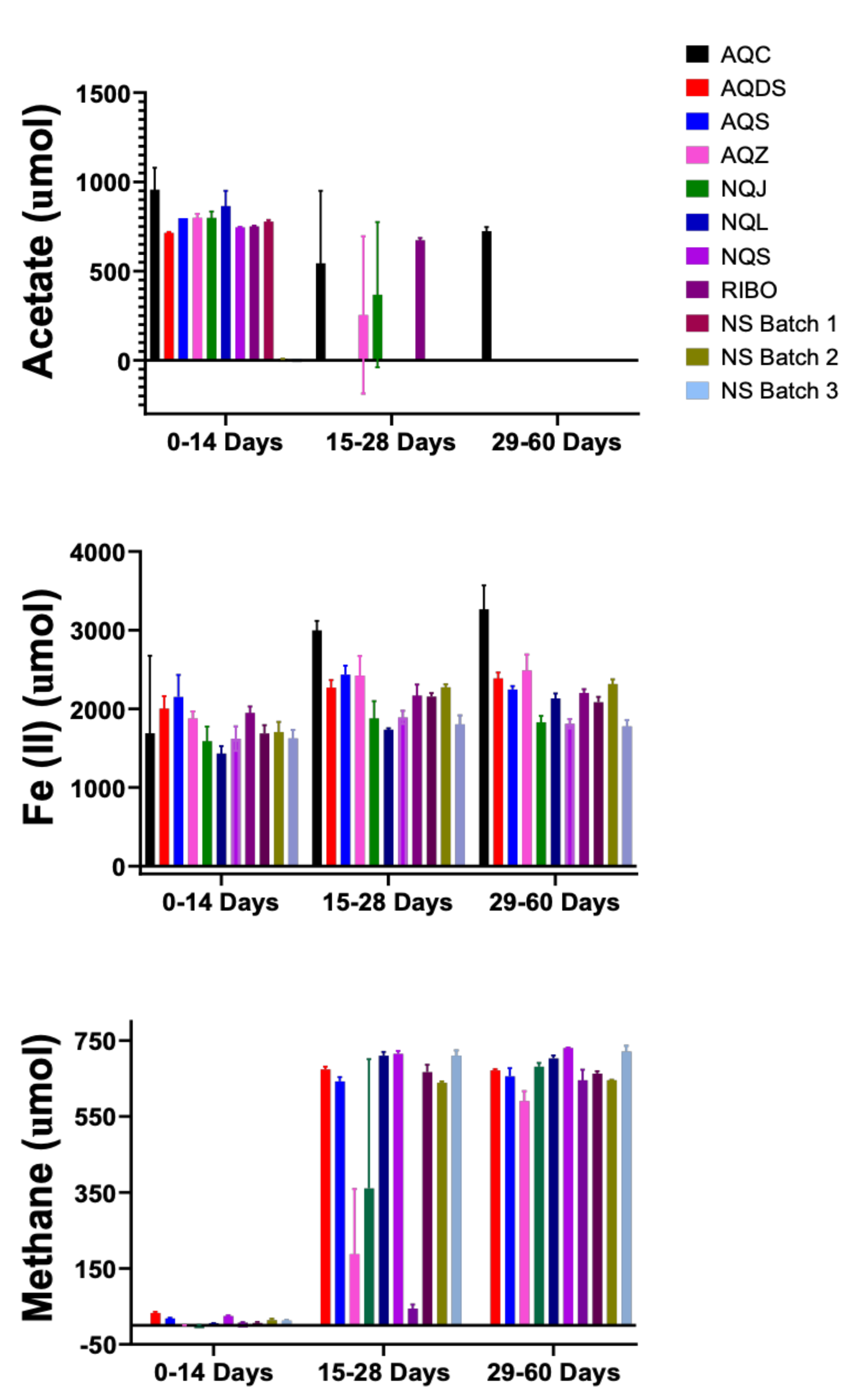
**

**Supplemental Figure S2.** Relatedness of all communities measured as Bray-Curtis on an NMDS.

**Supplemental Figure S3.** Stacked bar plots of the genera within the Geobacteraceae family for each shuttle treatment. N/A indicates not assigned at the genus level. The plot for each shuttle contains all three replicates per shuttle.


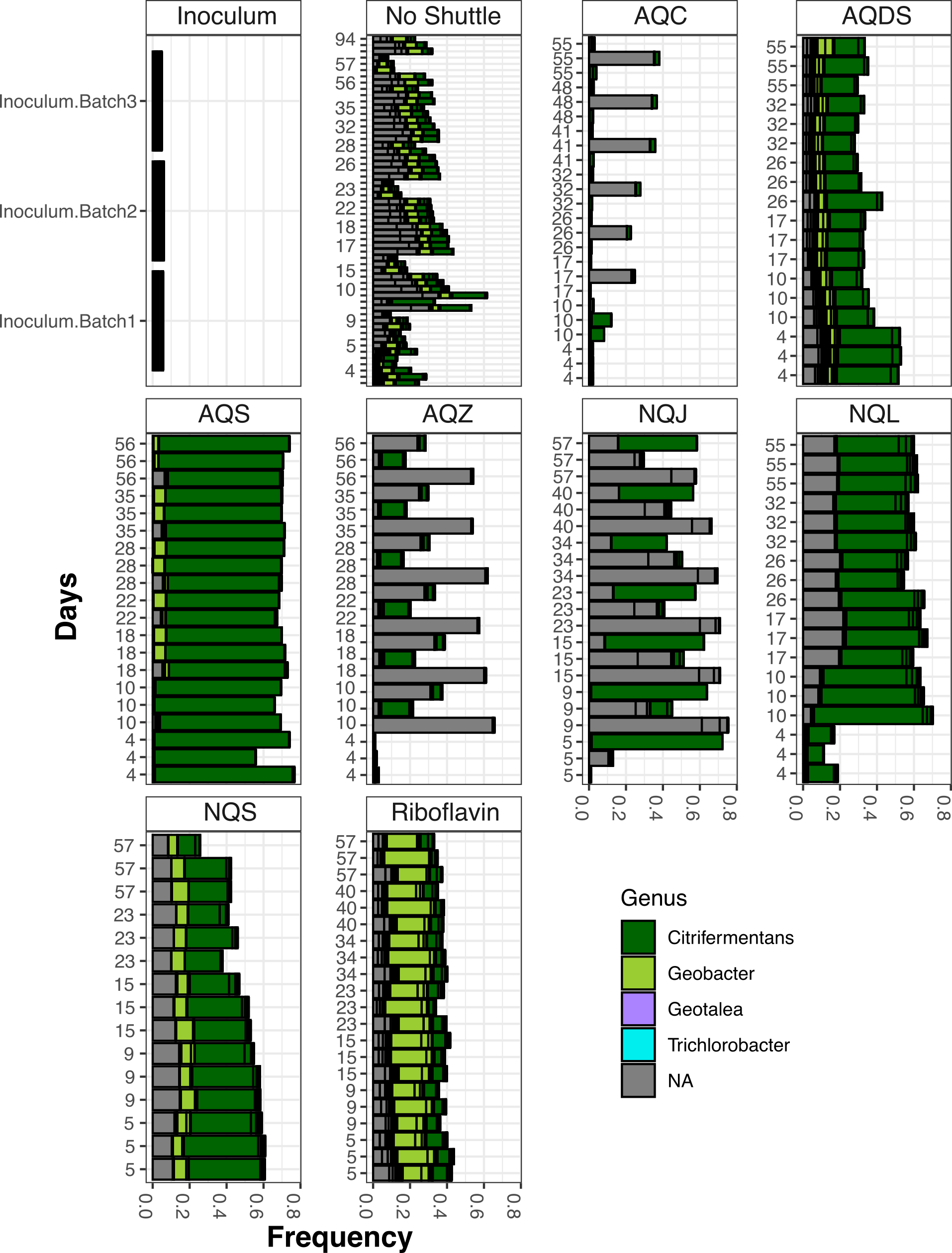


**Supplemental Figure S4.** Relative abundance of ASVs belonging to Halobacterota, Euryarchaeota, Crenarchaeota, or Methylomirabilota phyla.

**Supplemental Figure S5.** Conceptual diagram of our experimental system showing A) sediment collection using a corer from a submerged, Typha-dominated wetland B) inoculation with a wetland sediment slurry (from Batch 1, 2, or 3) of sealed bioreactors amended with a single, quinone-based electron shuttle compound, a source of Fe(III) oxide, and a defined mineral medium containing acetate C) monitoring the concentration of metabolic products and microbial community composition over time. Sediment used for batches 1, 2, and 3 were collected from the same wetland at different times.


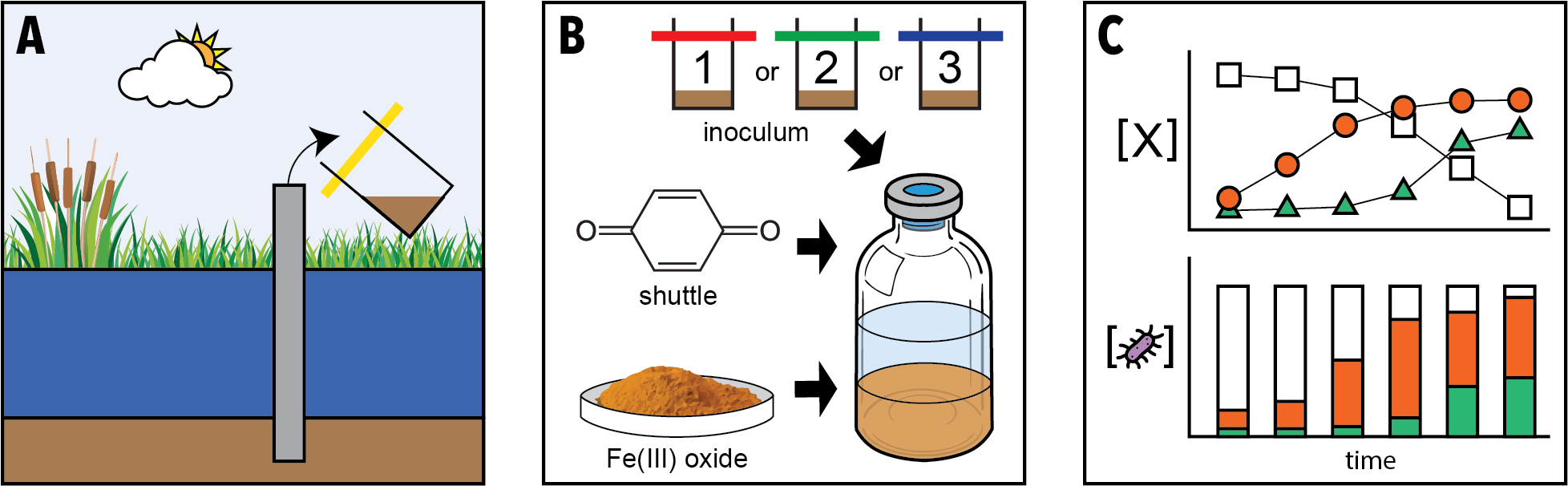

Supplement: Supplemental figures — Figures S1 to S5. [file aem.02222-24-s0001.docx]
